# Does Enriched Environment Improve Chronic Immobilization Stress-Induced Cognitive and Behavioral Changes in Adult Male Rats?

**DOI:** 10.1007/s10571-026-01712-y

**Published:** 2026-04-12

**Authors:** Mona A. Mohammed, Amira S. Ahmed, Fatma M. M. Salem, Eman S. H. Abd Allah

**Affiliations:** 1 Medical Physiology Department, Faculty of Medicine, Assiut University, Assiut, Egypt; 2 Histology and Cell Biology Department, Faculty of Medicine, Assiut University, Assiut, Egypt

**Keywords:** Chronic immobilization stress, Cognitive and behavioral changes, Enriched environment, Hippocampus

## Abstract

Chronic stress induces detrimental effects on cognition, behavior, and hippocampal integrity. An enriched environment (EE) has been shown to enhance learning and memory; however, its role against chronic immobilization stress (CIS)-induced alterations and the underlying mechanisms remain insufficiently explored. This study aimed to investigate the protective effects of EE on CIS-induced behavioral, molecular, and structural changes in the hippocampus of adult male rats. Thirty-two adult male Wistar albino rats were assigned to four groups: control, control + EE, CIS, and CIS + EE. Rats were subjected to CIS (4 h/day) followed by EE exposure (2 h/day) for 28 days. Behavioral assessments were conducted. Serum corticosterone levels, hippocampal brain-derived neurotrophic factor (BDNF), and mRNA expression of aquaporin-4 (AQP4) and glutamate receptors (GluA1 and GluA2) were evaluated. Histopathological, ultrastructural, and immunohistochemical (LC3) examinations were performed. EE significantly ameliorated CIS-induced cognitive and behavioral impairments and restored hippocampal histological and ultrastructural integrity. These effects were associated with reduced serum corticosterone levels, increased hippocampal BDNF levels, and upregulated expression of AQP4, GluA1, and GluA2 mRNA. These findings suggest that EE is a promising non-pharmacological strategy for mitigating stress-induced hippocampal dysfunction and cognitive decline.

## Introduction

Stress is now an unavoidable part of life due to the diverse range of pressures we encounter every day and the complexity of our modern living environment (Eladawy et al. 2025). Chronic stress refers to sustained contact to anxieties, causing the constant stimulation of the stress response system. This constant state of awareness can lead to major physical and mental health complications (Shchaslyvyi et al. 2024).

The primary driving force behind the neuroendocrine stress response is the hypothalamic–pituitary–adrenal axis (Varghese et al. 2023). Prolonged activity of the stress response system can affect brain interaction and organization, resulting in mood illnesses like anxiety and depression. Furthermore, excessive and prolonged elevation of cortisol levels can harm the hippocampus, a vital region for memory and knowledge, resulting in cognitive impairment (Montgomery and Gouvea 2024).

BDNF and its specific tyrosine kinase receptor (TrkB) are ubiquitous in brain regions related to mood disorders, including the hippocampus, prefrontal cortex, and amygdala (Machaalani and Chen 2018; Jiang et al. 2021). BDNF promotes neuronal survival and differentiation, and plays important roles in the recall of spatial memory and higher cognitive functions (Kang et al. 2019). Chronic stress and various other disorders, including depression and Alzheimer's disease, can deplete hippocampus BDNF and block the neuroprotective cell signaling cascades when BDNF binds to its receptor (Herhaus et al. 2023).

α-amino-3-hydroxy-5-methyl-4-isoxazolepropionic acid receptors (AMPARs) are ionotropic glutamate receptors that control synaptic plasticity (Partin 2015; Sun et al. 2022). There is an interplay between BDNF and AMPAR; increased AMPAR expression stimulates the secretion of BDNF, and BDNF increases the trafficking and the translation of GluA1 (Hartmann et al. 2001; Jourdi and Kabbaj 2013). GluA2 decreases the permeability of AMPARs to Ca^2+^ and thereby safeguards against excitotoxicity and neuronal death (Llorente et al. 2015).

Aquaporin 4 (AQP4), a unique protein that appears on the astrocyte end-feet, plays a key role in the blood–brain barrier (BBB) Functionality and glymphatic transport to clean the brain from waste products (Wei et al. 2019; Genel et al. 2021). AQP4 is exploited as a broad-spectrum therapeutic target for controlling or even reversing a range of complex causes of cognitive impairment or unexplained cognitive diseases (Wang et al. 2022).

Autophagy is considered a self-protective process that eliminates damaged organelles and clumped proteins. It may detect intracellular and extracellular stress and rapidly respond to cellular destruction (Eshraghi et al. 2022). The process is structured by a number of genes, including Beclin-1 and LC3-II, reliable biomarkers for measuring autophagy (Xu et al. 2022). Chronic stress can disrupt autophagy signaling in the hippocampus and frontal cortex, leading to alterations in neuronal structure (Ulecia-Morón et al. 2025).

An enriched environment (EE) is a giant cage filled with a range of equipment, such as wooden pieces of various sizes and shapes, which are changed on a regular basis and replaced with new objects for novelty (Khokhar et al. 2022). It enhances learning, memory, and synaptic plasticity through the reduction of corticosterone levels in offspring of the prenatal stress model (Guan et al. 2021), up-regulation of BDNF and TrkB in a moderate exercise combined with the enriched environment model (Xu et al. 2021), axonal sprouting, synaptogenesis, neurogenesis, and vascular remodeling in the post-stroke rehabilitation model (McDonald et al. 2018). Additionally, EE alleviates cognitive impairment and depressive-like behaviors resulting from chronic stress through the initiation of autophagy in the hippocampal tissue (Xu et al. 2022).

Thus, the present study aims to investigate the effect of EE in reducing CIS-induced cognitive and behavioral changes through the evaluation of BDNF, aquaporin 4, AMPA glutamate receptors (GluA1, GluA2) levels, and LC3 as possible mechanisms. Additionally, we assess the extent to which EE can improve the histopathological and ultrastructural appearance of hippocampal tissue in CIS-exposed rats.

## Materials and Methods

### Animals and Experimental Design

#### Sample Size Calculation

Calculation of sample size in an animal study through a power analysis using the KISS approach shows that the sample size of 8 rats/group (8*4 groups) has an 80% power to detect a hypothesized standardized effect size of 1.5 SDs between the control and treated group to identify the effect of enriched environment in attenuating behavioral changes and cognitive impairment induced by chronic restraint stress in adult male rats, assuming a 5% significance level and a two-sided test (Kilkenny and Altman 2010; Festing 2018).

A total of thirty-two adult male Wistar Albino rats weighing approximately 180–200 g, obtained from the Animal House of the Faculty of Medicine, Assiut University, were recruited in this study. Rats were housed in plastic cages (4 rats per cage) and kept on a natural light–dark cycle at room temperature and had free access to standard laboratory rodent chow. Prior to conducting the experiment, ethical approval for the study was obtained from the ethical committee of the Faculty of Medicine -Assiut University (IRB approval no: 04-2023-200264) that adheres to the National Institutes of Health's regulations for the treatment and care of research animals.

The investigators were blinded to the group assignments during the experimental procedures and the subsequent statistical analysis to ensure unbiased data collection and interpretation.

After adaptation for one week, rats were randomly assigned into four groups, with eight rats in each 8a.m. to 12p.m. The control enriched environment group (CEE) had no access to food or water from 8a.m. to 12p.m. and then was exposed to EE for 2 h/day (from 12p.m. to 2p.m.) for 28 days. The chronic immobilization stress group (CIS) was exposed to daily immobilization stress for 4 h/day (from 8a.m. to 12p.m.) for 28 days. The chronic immobilization stress + enriched environment group (CIS + EE) was exposed to daily immobilization stress for 4 h/day (from 8a.m. to 12p.m.) followed by 2 h EE/day (from 12p.m. to 2p.m.) for 28 days.

### Chronic Immobilization Stress (CIS) Induction Protocol

Each rat in the CIS and CIS + EE groups was placed into a well-ventilated plastic tube suitable to its size that limited body movement except for slight head movement (Jiang et al. 2021) for 4 h/day for 28 days (Vanisree and Thamizhoviya 2021).

### Enriched Environment (EE) Protocol

**Fig. 1:**
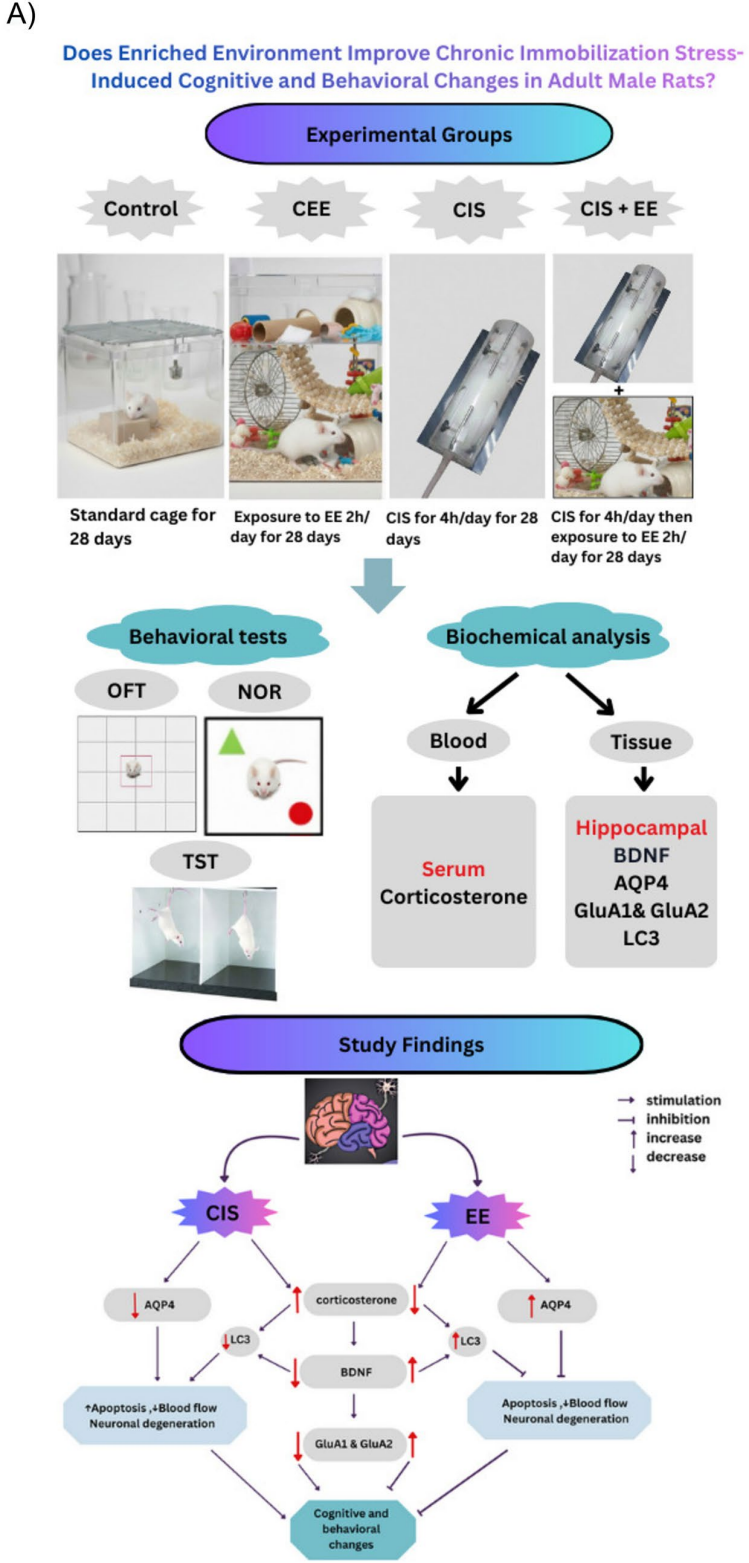

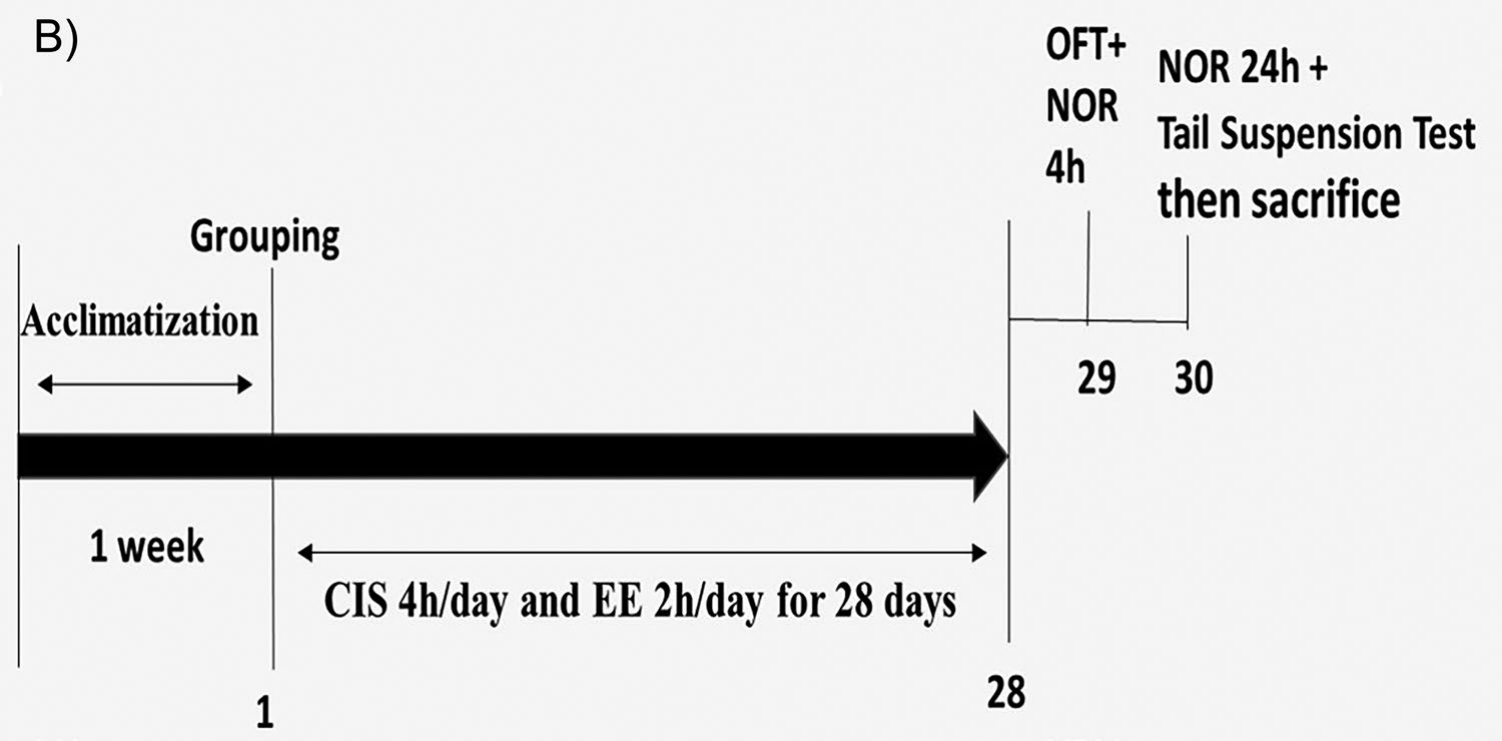
**A** Graphical abstract of study. After 28 days of CIS and EE protocols, rats were evaluated using behavioral and biochemical assessments. Results show that exposure to EE reversed cognitive and behavioral changes via decreased corticosterone level, increased BDNF level, increased AQP4 expression, increased GluA1, GluA2 expression, and increased LC3 level. **B** Time course of the experiment. *CIS* Chronic immobilization stress, *EE* Enriched environment, *NOR* Novel object recognition test, *OFT* Open field test

Rats from CEE and CIS + EE groups were placed in a large novel cage. To preserve the novelty, the rats were permitted to explore and play with new objects for two hours every day for 28 days. (Vanisree and Thamizhoviya 2021). The graphical abstract and the time course of the experiment are shown in Fig. 1A and B respectively. At the end of 28 days, the behavioral tests were done on all rats of the study groups.

### Behavioral Tests

I- Open field test OFT): Rats' exploration and locomotion were tested using the open field test. Rats were subjected to a 5 min acclimatization in an open area of 60 × 60 × 30 cm. A day after acclimatization, each rat was located individually in the center of the apparatus for 10 min, and the number of lines crossed and central square entries were counted, further measuring the time spent in the central square (Walsh and Cummins 1976; Bahi 2016).

II- Novel object recognition test (NOR): Rats' memory was assessed by this recognition memory test. The test is composed of three phases: adaptation for 3 min in an empty open box of 60 × 60 × 30 cm, familiarization for another 3 min in the same apparatus containing duplicated objects (green cones considered as familiar objects), and testing for 3 min.

To assess intermediate and long-term memory during the testing phase, each rat was placed in the same apparatus enclosing one familiar object (green cone) and one novel object (red round object), after familiarization via four hours and 24 h, respectively. Normal rats usually spend most of the three minutes examining the novel object.

The alteration in assessment time for the novel (TN) and familiar (TF) objects was calculated and divided by the total examination time for both objects and is considered as the discrimination index (DI); [DI = (TN − TF)/(TN + TF)] (Ennaceur and Delacour 1988; Antunes and Biala 2012; Abd Allah et al. 2014).

III- Tail Suspension Test (TST): For five minutes, rats were suspended by their tails. The rat's tail was attached to the tube ten centimeters above the ground. During the testing period, the rats attempted to escape and reach the ground, and their immobility time was noted and calculated (Steru et al. 1985; Cryan et al. 2005; Vanisree and Thamizhoviya 2021).

### Sample Collection

At the end of the 28-day experimental period, following completion of the behavioral assessments, blood samples were collected from the retro-orbital venous plexus. After collection, the samples were centrifuged, and supernatant sera were collected and retained at − 20 °C until used for corticosterone determination. At the end of the experimentation, the rats were anesthetized and humanely sacrificed. The skull was opened, and the brain was cautiously and rapidly removed, washed with cold phosphate buffered saline (PBS), and separated into two hemispheres; the right hemisphere was for histopathological evaluation, and the left hippocampus was dissected from the other hemisphere and divided into two halves, then immediately frozen in liquid nitrogen and lastly stored at − 80 °C until used for BDNF and qPCR determination.

### Biochemical Analysis

Serum corticosterone level was determined using an ELISA kit (Cat. No. REF: CAN-C-270, Diagnostics Biochem Canada Inc., USA). Adhering to the manufacturer's guidelines. The hippocampal tissue was homogenized in fresh lysis buffer (PBS), and the supernatant was collected. The protein concentration of the supernatant was assessed using bovine serum albumin (Cat. No. REF: 210 001, Egyptian Company for Biotechnology, Cairo, Egypt) according to the Lowry method (1951). After that, the BDNF level was determined using the rat BDNF ELISA Kit (Cat. No. ELK5459, ELK Biotechnology, Wuhan, P.R.C.) in accordance with the documentation (Lowry et al. 1951; Abdel-Lah et al. 2025).

### Quantitative Real-Time PCR (q-PCR) Analysis

Total RNA was extracted from the left hippocampus using the Qiagen RNeasy Mini Kit (Cat. no. 74104, Qiagen, Germantown, MD, USA) and then reverse transcribed using the Applied Biosystems™ High-Capacity cDNA Reverse Transcription Kit (Cat. no. 4368814, Applied Biosystems, Foster City, CA, USA) following the instructions provided by the manufacturer. Quantitative PCR analysis was performed using SYBR Green Super Mix (Cat. no. 204143, Applied Biosystems, and Foster City, CA, USA) and a 7500 Fast Real-Time PCR System. The relative expression levels of AQP4, GluA1 & GluA2 mRNA versus GAPDH as a housekeeping gene are shown in the Table 1 was calculated using the 2^−ΔΔ*ct*^ method and was represented as the fold change relative to the control group; its value is 1 (Molina-Navarro et al. 2013).

**Table 1:** The primers sequences for GAPDH, AQP4, and GluA1 and GluA2

Primer	Sequence (5′–3′)	References
GAPDH	F: 5′-ATGGGAGTTGCTGTTGAAGTCA-3′ R: 5′-CCGAGGGCCCACTAAAGG-3′	Miyatake et al. (2006)
AQP4	F: 5′-TGAATCCAGCTCGATCCTTTG-3′ R: 5′-TATCCAGTGGTTTTCCCAGTTTC-3′	Shi et al. (2021)
GluA1	F: 5′-TCCCCAACAATATCCAGATAGGG-3′ R: 5′-AAGCCGCATGTTCCTGTGATT-3′	Basha et al. (2023)
GluA2	F: 5′-AATGGACGTGTTATGACTCCAGA-3′ R: 5′-CTGACATTCATTCCCATGCCA-3′	Basha et al. (2023)

### Histopathological and Immunohistochemical Examination

The right hippocampal tissue from all experimental animals after scarification was retained in 10% formalin and processed as formalin-fix paraffin-embedded tissue sections for histopathological and immunohistochemical examinations. The general histological architecture was evaluated under light microscopy by cutting the paraffin-embedded tissue sections into 5–7 μm thickness using the microtome, then they were stained using Hematoxylin and Eosin (H&E) stain to examine the overall hippocampal tissue structure (Alsemeh et al. 2020).

The paraffin hippocampal formalin-fixed sections were mounted on positive charged slides, subjected to dewaxing in xylene, and rehydrated via a graded alcohol series to water. To perform antigen retrieval, the tissue was heated in a 10-um citrate buffer for 10 min. Then, the sections were applied with the primary antibody for light chain 3 A and B (LC3A/LC3B) antibody for the detection of autophagosomes (Catalog # PA1-16931) purchased from Thermo Scientific, USA. The primary antibody was a rabbit polyclonal antibody using the avidin–biotin-peroxidase complex technique. The reaction was seen using DAB-hematoxylin staining and examined with a light microscope (Cattoretti et al. 1993). No primary antibody control sections were prepared using phosphate buffer saline (PBS). Positive control sections were from the colon and cerebral cortex (according to data provided by the antibody manufacturer). LC3-positive immunostained cells appeared with brown cytoplasmic reactions and blue nuclei.

### Electron Microscopy Examination

A minimum of 24 h was required to right hippocampal tissues that remained to be used for ultrastructural examination in 5% glutaraldehyde, then continue for preparation of semi-thin Sects. (0.5–1 μm thick) that were stained with toluidine blue and examined by light microscope. From selected areas of semi-thin sections, ultra-thin sections (500–800 nm) were cut, collected on copper grids, and contrasted with uranyl acetate and lead citrate (Rosińczuk et al. 2015). They were examined and photographed by the transmission electron microscope JEOL (JEM-100 CX11, TOKYO, JAPAN) at 80 kV in Assiut University-Electron Microscope Unit.

### Morphometric Study

The hippocampal tissue sections after immunostaining were photographed and documented with an Olympus Color View camera and analysis software (Soft Imaging System) at the Histology Department, Faculty of Medicine, at Assiut University. Finally, the number of LC3A&B immune-positive cells from eight slides of each group were randomly inspected, and measurements were taken from 10 randomly selected non-overlapping fields at a magnification of 1000 from each group's animals (Helal et al. 2020).

### Statistical Analysis

Data were assessed using SPSS version 26 (SPSS Inc., Chicago, USA). Normality of the data was judged by means of the Shapiro–Wilk test. Data were normally distributed and analyzed using one-way ANOVA followed by Tukey's post-hoc test. Then the results were presented as mean ± standard deviation (SD), and *P* value < 0.05 was considered statistically significant. The correlation between measured parameters was done using Pearson's correlation.

## Results

### Behavioral Test Results

#### Open Field Test (OFT)

**Fig. 2:**
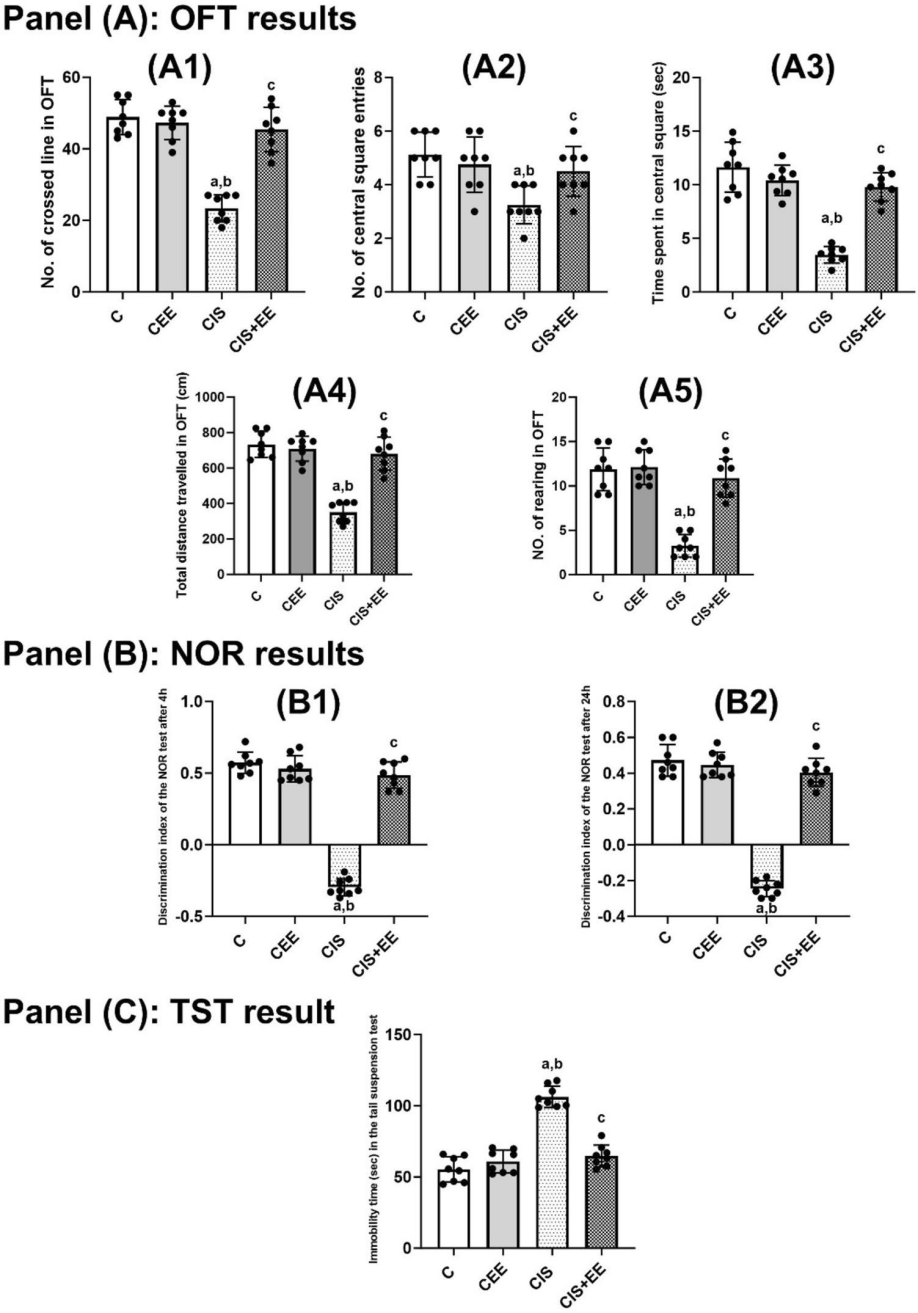
Behavioral tests results. Panel **A** Open field test in all groups studied. A1: No. of crossed lines, A2: No. of central square entries, A3: time spent in central square (sec), A4: distance travelled (cm), and A5: No. of rearing. Panel **B** Discrimination index of the NOR test in all groups studied. B1: after 4 h and B2: after 24 h. Panel **C** Immobility time (sec) in the tail suspension test in all groups studied. Data are expressed as mean ± SD (n = 8/group). *C* control group, *CEE* control enriched environment group, *CIS* chronic immobilization stress group, *CIS* + *EE* chronic immobilization stress + enriched environment group. ^a^indicates *p* < 0.05 vs. the control group, ^b^indicates *p* < 0.05 vs. the CEE group, and ^c^indicates *p* < 0.05 vs. the CIS group. One-way ANOVA test followed by Tukey post-hoc test was used for statistical analysis

The number of crossed lines, the number of central square entries, time spent in the central square, total distance travelled, and number of rearing showed homogeneity of variances (Brown-Forsythe test, *F*_(3, 28)_ = 0.50, *p* = 0.685, *F*_(3, 28)_ = 0.362, *p* = 0.781, *F*_(3, 28)_ = 2.549, *p* = 0.076, *F*(3, 28) = 0.5, *p* = 0.685, F(3, 28) = 1.25, p = 0.3; respectively). One-way ANOVA revealed a significant difference in the number of crossed lines, the number of central square entries, time spent in the central square, total distance travelled, and number of rearing between the studied groups (*F*_(3, 28)_ = 46.60, *p* < 0.001, *F*_(3, 28)_ = 6.76, *p* = 0.001, *F*_(3, 28)_ = 43.78, *p* < 0.0001, *F*(3, 28) = 46.60, *p* < 0.0001, F(3, 28) = 35.61, p < 0.0001; respectively). There were no significant differences in the number of crossed lines, the number of central square entries, time spent in the central square, total distance travelled, and number of rearing between the control group and CEE group (48.88 ± 4.91, 5.13 ± 0.83, 11.63 ± 2.32, 733.1 ± 73.68, 11.88 ± 2.42 vs. 47.25 ± 4.68, 4.75 ± 1.04, 10.41 ± 1.42, 708.8 ± 70.24, 12.13 ± 1.96, p value = 0.913, 0.831, 0.412, 0.913, 0.99; respectively). The CIS group showed a statistically significant decline in the test results in comparison to the control and CEE groups (23.38 ± 3.78 vs. 48.88 ± 4.91 and 47.25 ± 4.68, 3.25 ± 0.71 vs. 5.13 ± 0.83 and 4.75 ± 1.04, 3.35 ± 0.77 vs. 11.63 ± 2.32 and 10.41 ± 1.42, 350.6 ± 56.66 vs. 733.1 ± 73.68 and 708.8 ± 70.24, 3.25 ± 1.28 vs. 11.88 ± 2.42 and 12.13 ± 1.96, p value < 0.0001; respectively); indicating reduced locomotor activity and depressed behavior. However, rats exposed to CIS + EE displayed a significant upsurge in the number of previous findings with statistically significant differences compare to the CIS group (45.83 ± 6.19, 4.50 ± 0.93, 9.80 ± 1.32, 680.6 ± 92.79, 10.88 ± 2.17 vs. 23.38 ± 3.78, 3.25 ± 0.71, 3.35 ± 0.77, 350.6 ± 56.66, 3.25 ± 1.28, p value < 0.0001; respectively); indicating EE improved locomotor activity as shown in Fig. 2, Panel A.

#### Novel Object Recognition Test (NOR)

The discrimination index after 4 h and 24 h showed homogeneity of variances (Brown-Forsythe test, *F*_(3, 28)_ = 0.832, *p* = 0.487, *F*_(3, 28)_ = 0.750, *p* = 0.531; respectively). One-way ANOVA revealed a significant difference in the discrimination index after 4 h and 24 h between the studied groups (*F*_(3, 28)_ = 211.5,* p* < 0.0001, *F*_(3, 28)_ = 180.7, *p* < 0.0001; respectively). The discrimination index after 4 h and 24 h between the control and CEE groups showed no significant alterations (0.57 ± 0.07, 0.47 ± 0.09 vs. 0.53 ± 0.09, 0.45 ± 0.07, p value = 0.719, 0.9; respectively). Compared to the control and CEE groups, the CIS group had a significant decrease in the discrimination index after 4 h and 24 h (0.57 ± 0.07, 0.53 ± 0.09 vs. − 0.30 ± 0.06 and 0.47 ± 0.09, 0.45 ± 0.07 vs. − 0.25 ± 0.04, p value < 0.0001; respectively) indicating reduced intermediate and long-term memory. Meanwhile, the CIS + EE group presented a statistically significant increase in this index in comparison to the CIS group (0.49 ± 0.09, 0.40 ± 0.08 vs. − 0.30 ± 0.06, − 0.25 ± 0.04, p value < 0.0001; respectively) (Fig. 2, Panel B).

#### Tail Suspension Test (TST)

Data showed homogeneity of variances (Brown-Forsythe test, *F*_(3, 28)_ = 0.331, *p* = 0.803). One-way ANOVA revealed a significant difference in the immobility time between the studied groups (*F*_(3, 28)_ = 68.16,* p* < 0.0001). The immobility time revealed no significant difference between the control and CEE groups (55.28 ± 8.77 vs. 60.85 ± 8.00, *p* value = 0.513). The CIS group had a significant increase in the immobility time compared to the control and CEE groups (106.30 ± 7.50 vs. 55.28 ± 8.77 and 60.85 ± 8.00, *p* value < 0.0001; respectively). In contrast, rats in the CIS + EE group showed a significant decrease in the immobility time in comparison to the CIS group (64.80 ± 7.61 vs. 106.30 ± 7.50, *p* value < 0.0001) as can be seen in Fig. 2, Panel C.

### Biochemical Results

#### Serum Corticosterone Level (ug/dl)

**Fig. 3:**
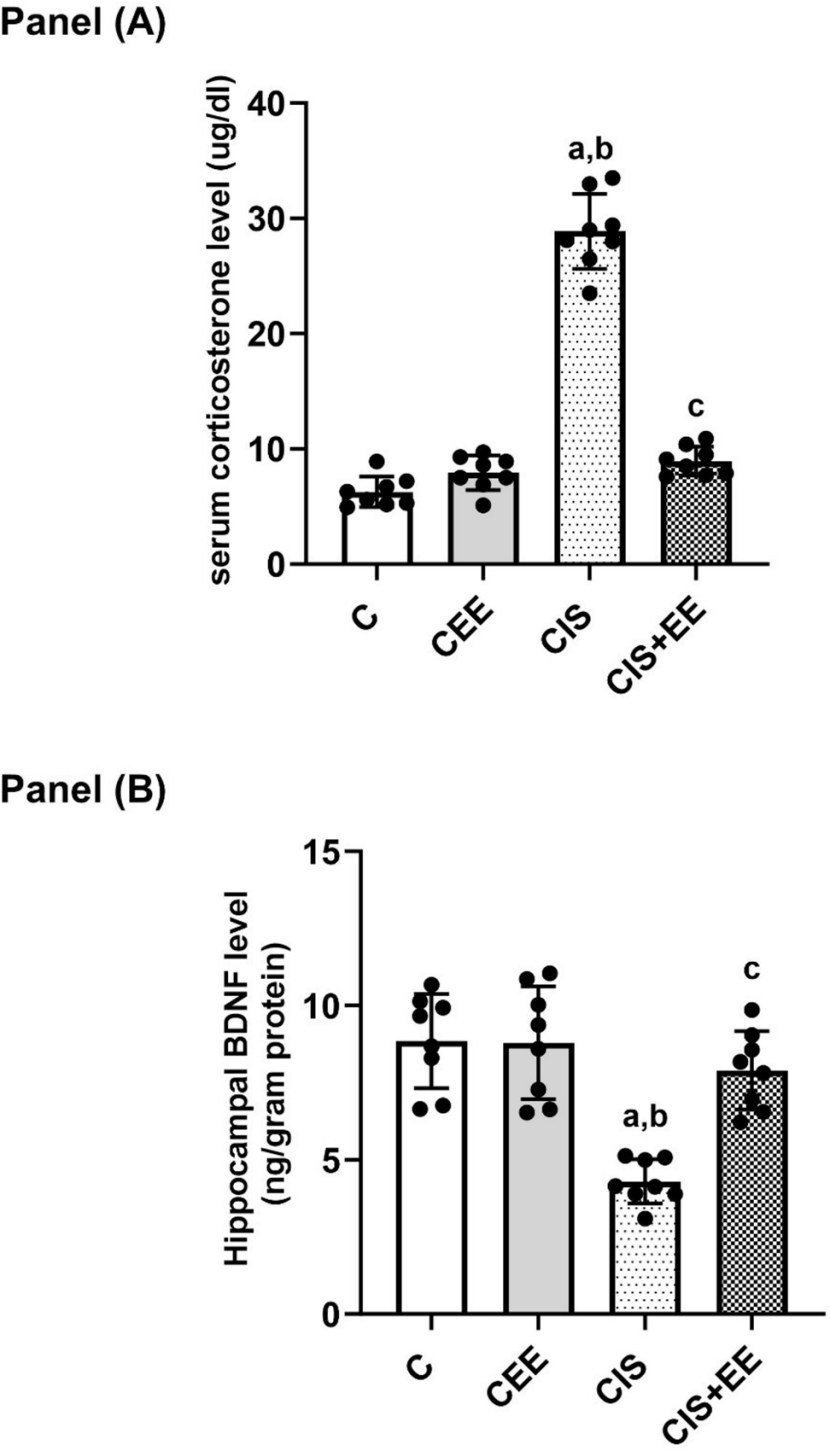
Panel **A** Serum corticosterone level (ug/dl) in all groups studied. Panel **B** Hippocampal Brain-Derived Neurotrophic Factor (BDNF) level (ng/gram protein) in all groups studied. Data are expressed as mean ± SD (n = 8/group). *C* Control group, *CEE* control enriched environment group, *CIS* chronic immobilization stress group, *CIS* + *EE* chronic immobilization stress + enriched environment group. ^a^indicates p < 0.05 vs. the control group, ^b^indicates p < 0.05 vs. the CEE group, and ^c^indicates p < 0.05 vs. the CIS group. One-way ANOVA test followed by Tukey post-hoc test was used for statistical analysis

Data showed homogeneity of variances (Brown-Forsythe test, *F*_(3, 28)_ = 2.13, *p* = 0.119). One-way ANOVA revealed a significant difference in serum corticosterone level between the studied groups (*F*_(3, 28)_ = 223.1, *p* < 0.0001). The serum corticosterone level between the control and CEE groups are quite near, with no significant difference (6.27 ± 1.32 vs. 7.94 ± 1.50, *p* value = 0.365). Serum corticosterone level increased significantly in the CIS group compared to the control and CEE groups (28.88 ± 3.26 vs. 6.27 ± 1.32 and 7.94 ± 1.50, *p* value < 0.0001; respectively). However, in the CIS + EE group, we observed a significantly reduced serum corticosterone level, which was statistically different compared to the CIS group (8.95 ± 1.25 vs. 28.88 ± 3.26, *p* value < 0.0001), as presented in Fig. 3, Panel A.

#### Hippocampal Brain-Derived Neurotrophic Factor (BDNF) Level (ng/gram Protein)

Data showed homogeneity of variances (Brown-Forsythe test, *F*_(3, 28)_ = 2.83, *p* = 0.057). One-way ANOVA revealed a significant difference in hippocampal BDNF level between the studied groups (*F*_(3, 28)_ = 19.07, *p* < 0.0001). There was no significant difference in hippocampal BDNF level between the control and CEE groups (8.85 ± 1.53 vs. 8.80 ± 1.83, *p* value = 0.999). Hippocampal BDNF level decreased significantly in the CIS group compared to the control and CEE groups (4.30 ± 0.72 vs. 8.85 ± 1.53 and 8.80 ± 1.83, *p* value < 0.0001; respectively). Moreover, rats exposed to EE following chronic immobilization stress in the CIS + EE group showed a significant increase in BDNF level in the hippocampus in comparison to the CIS group (7.90 ± 1.27 vs. 4.30 ± 0.72, *p* value < 0.0001) as shown in Fig. 3, Panel B.

### PCR Results

#### Fold Expression of Hippocampal AQP4 mRNA

**Fig. 4:**
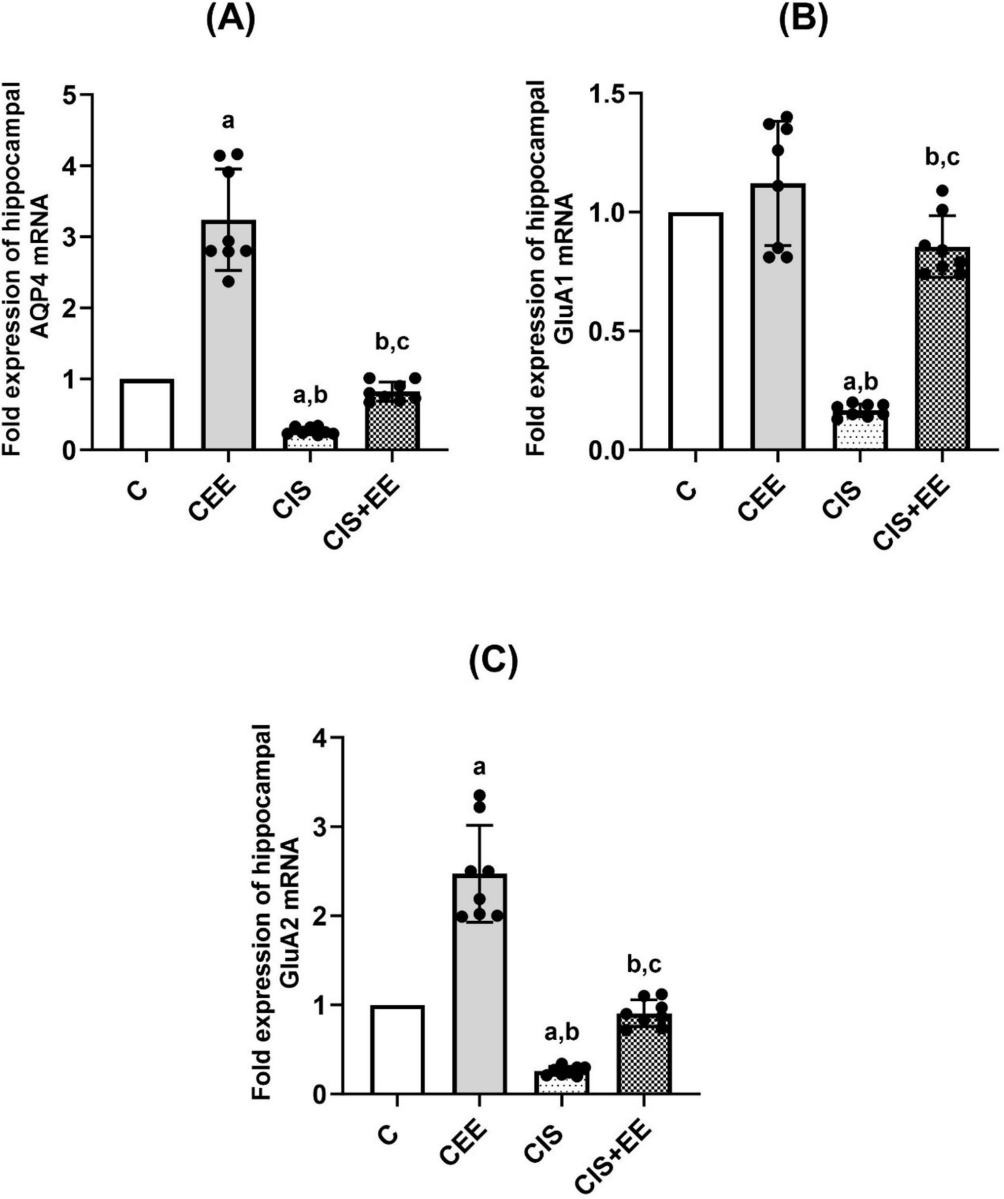
The fold expression in all groups studied. **A** Fold expression of AQP4 mRNA, **B** fold expression of GluA1 mRNA, and **C** fold expression of GluA2 mRNA. Data are expressed as mean ± SD (n = 8/group). *C* control group, *CEE* control enriched environment group, *CIS* chronic immobilization stress group, *CIS* + *EE* chronic immobilization stress + enriched environment group. ^a^indicates p < 0.05 vs. the control group, ^b^indicates p < 0.05 vs. the CEE group, and ^c^indicates p < 0.05 vs. the CIS group. One-way ANOVA test followed by Tukey post-hoc test was used for statistical analysis

One-way ANOVA revealed a significant difference in the fold expression of AQP4 mRNA between the studied groups (*F*_(3, 28)_ = 103.9, *p* < 0.0001). Rats in the CEE group had significantly higher AQP4 mRNA compared to the control group (3.24 ± 0.71 vs. 1, *p* value < 0.0001). Figure 4A showed that the fold expression of AQP4 mRNA decreased significantly in the CIS group in comparison to the control and CEE groups (0.27 ± 0.05 vs. 1 and 3.24 ± 0.71, *p* value < 0.01, 0.0001; respectively). While, rats in CIS + EE showed a significantly higher expression of AQP4 mRNA compared to the CIS group (0.82 ± 0.14 vs. 0.27 ± 0.05, *p* value < 0.05).

#### Fold Expression of Hippocampal GluA1 mRNA

One-way ANOVA revealed a significant difference in the fold expression of GluA1 mRNA between the studied groups (*F*_(3, 28)_ = 67.86, *p* < 0.0001). Rats in the CEE group tend to have an increased GluA1 mRNA compared to the control; however, this increase is not significant (1.12 ± 0.26 vs. 1, *p* value = 0.375). Figure 4B noticed that the fold expression of GluA1 mRNA reduced significantly in the CIS group in comparison to the control and CEE groups (0.17 ± 0.03 vs. 1 and 1.12 ± 0.26, *p* value < 0.0001; respectively). Exposure to EE following CIS caused a significant increase in the expression of GluA1 mRNA in CIS + EE compared to the CIS group (0.86 ± 0.13 vs. 0.17 ± 0.03, *p* value < 0.0001).

#### Fold Expression of Hippocampal GluA2 mRNA

One-way ANOVA revealed a significant difference in the fold expression of GluA2 mRNA between the studied groups (*F*_(3, 28)_ = 87.1, *p* < 0.0001). Rats in the CEE group had a significantly higher GluA2 mRNA compared to the control group (2.47 ± 0.54 vs. 1, *p* value < 0.0001). Q-PCR showed that the fold expression of GluA2 mRNA reduced significantly in the CIS group in comparison to the control and CEE groups (0.26 ± 0.05 vs. 1 and 2.47 ± 0.54, *p* value < 0.0001; respectively), as can be seen in Fig. 4C. While, rats in CIS + EE showed a significantly higher expression of GluA2 mRNA compared to the CIS group (0.91 ± 0.15 vs. 0.26 ± 0.05, *p* value < 0.001).

### Histopathological Results

#### Cytoarchitectural Analysis of the Hippocampus Using H&E Staining

In the control group (C), examination of parasagittal sections of rats' hippocampi revealed the classical structure of the hippocampus that was composed of the hippocampus proprius (CA) and the dentate gyrus (DG). The CA was further subdivided into four areas (CA1, CA2, CA3, and CA4) consisting of 1-Stratum pyramidal (SP). 2- Stratum oriens 3- Stratum radiatum (SR) 4- Stratum Lacunosum 5- stratum moleculare. The DG was detected as a coiled assembly with an open concave part directed to the hippocampus proprius. The DG consisted of three layers: stratum molecular, stratum granulae, and stratum multiforme.

**Fig. 5:**
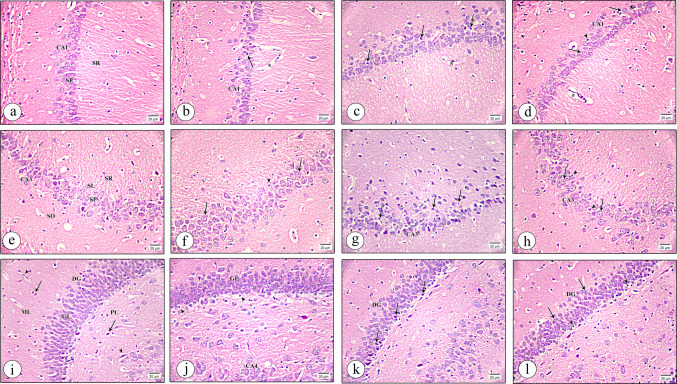
A photomicrograph of the hippocampus tissue sections stained with hematoxylin and eosin (H&E) at × 400 magnification. CA1 region **a** Control group exhibiting medium-sized pyramidal neurons in stratum pyramidal (SP) with their dendrites projecting into stratum radiatum (SR). **b** CEE group showing that most pyramidal neurons and few darkly stained shrunken cells (arrows) with empty spaces around the blood capillaries (asterisk). **c** CIS group revealing many degenerated neuronal cells (arrows) and the empty spaces (asterisk). **d** Many pyramidal neurons (arrowheads), some dark, shrunken cells (arrows) and wide empty spaces (asterisk) are observed in CIS + EE. CA3 region **e** Control group showing stratum pyramidal (SP), the stratum oriens (SO), stratum lucidum (SL), and stratum radiatum (SR). **f** CEE group observing a few dark, shrunken pyramidal cells (arrowheads), and many large pyramidal neurons (arrows) are seen. **g** CIS group displaying most pyramidal neurons are shrunken, and deeply stained (arrows). **h** CIS + EE group exhibiting many large pyramidal neurons with pale nuclei and basophilic cytoplasm (arrowheads). Some dark, shrunken, pyramidal neurons with irregular outlines (arrows) are also noticed. Dentate gyrus (DG) region **i** Control group showing closely packed granular neurons in the granular cell layer (GL), the scattered cells (arrow) and the blood capillaries (arrowhead) in the outer molecular (ML) and the inner pleomorphic (PL) layers. **j** CEE group presenting closely packed granular neurons in GL. Notice a few deeply stained nuclei and vacuolated cytoplasm at the basal zone of GL (arrowheads) and part of CA4 cells. **k** CIS group revealing many degenerated granular neurons (arrows). **l** Closely packed granule neurons with pale vesicular nuclei (arrow), and some dark granule cells (arrowheads) especially at the basal zone are noticed in CIS + EE group

The CA1 region showed medium-sized pyramidal neurons closely packed in stratum pyramidal (SP) with their long prominent dendrites projecting into stratum radiatum (SR) (Fig. 5a). The CA3 area showed stratum pyramidal (SP) that enclosed large pyramidal neuronal cells with pale nuclei and basophilic cytoplasm (Fig. 5e). The dentate gyrus (DG) field showed pale rounded nuclei of granular neurons that were closely packed in the granular cell layer (GL). In addition to scattered cells, the blood capillaries in the outer molecular (ML) and the inner pleomorphic (PL) layers were observed (Fig. 5i).

The hippocampal tissue showed more or less normal configuration and architecture in the CEE group that was practically indistinguishable from the sections of the normal group, with few darkly stained cells in the CA1 region (Fig. 5b), few dark, shrunken pyramidal cells surrounded by spaces in the CA3 area (Fig. 5f), and also a few deeply stained nuclei and vacuolated cytoplasm at the basal zone of granular neurons in the DG were observed (Fig. 5j).

While in the CIS-exposed group, there was deterioration in the hippocampal tissue structure formed of the CA1 field of Ammon's horn, which revealed many darkly stained, shrunken neuronal cells separated by narrow and spaced blood capillaries (Fig. 5c). Most pyramidal neurons were shrunken with deeply stained nuclei in the CA3 area (Fig. 5g). Many granular neurons had deeply stained nuclei and vacuolated cytoplasm in the DG region (Fig. 5k).

In contrast, in the CIS + EE group, we noticed many pyramidal neurons contained pale nuclei with some dark, shrunken pyramidal neurons in the CA1 area (Fig. 5d). The CA3 field of the same group showed many intact large pyramidal neurons and some dark, shrunken pyramidal neurons with irregular outlines (Fig. 5h). In addition to that, many closely packed granule neurons with pale vesicular nuclei in the DG area which were separated by few empty spaces, surrounded some dark granule cells, especially at the basal zone of the DG region were seen (Fig. 5l).

#### Immunohistochemical Staining with Morphometrically Quantitative Estimation of LC3 Antibody Results

**Fig. 6:**
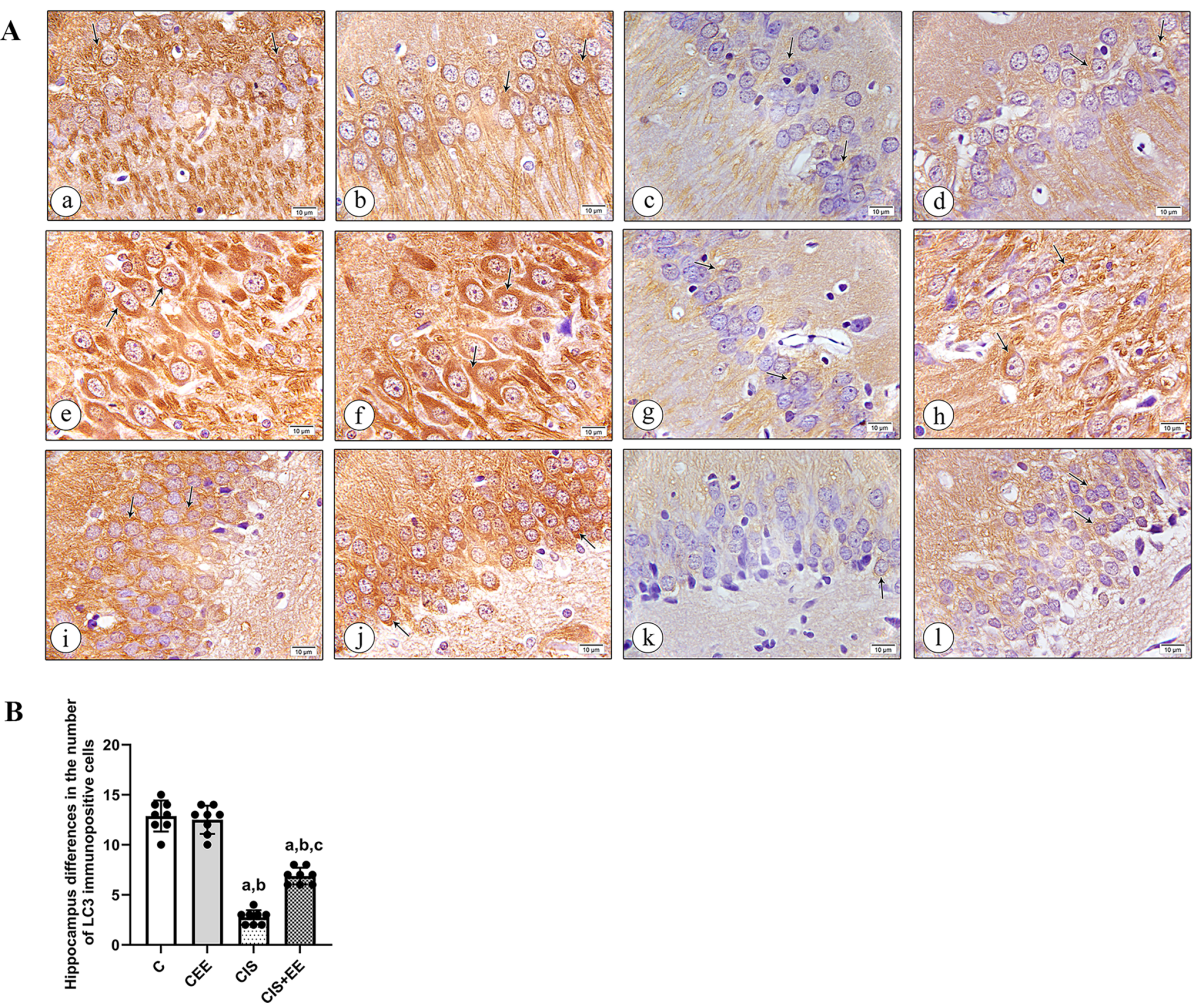
**A** A photomicrograph of hippocampus tissue sections immunohistochemically stained with LC3 (× 1000). Control group showing numerous positively LC3 immuno-stained cells (arrows) in all layers of the CA1 (**a**), CA3 (**e**), and DG region (**i**). CEE group revealing many positive LC3 immuno-stained cells with strong cytoplasmic reactions (arrows) in all layers of the CA1 (**b**), CA3 (**f**), and DG region (**j**). CIS group displaying a few positive LC3 immuno-stained cells (arrows) in all layers of the CA1 (**c**), CA3 (**g**), and DG region (**k**). CIS + EE group exhibiting some positive LC3 immuno-stained cells (arrows) in all layers of CA1 (**d**), CA3 (**h**), and DG area (**l**). **B** The mean differences in the number of LC3 immunopositive cells in the hippocampi tissue sections in all groups studied. Data are expressed as mean ± SD (n = 8/group). *C* control group, *CEE* control enriched environment group, *CIS* chronic immobilization stress group, *CIS* + *EE* chronic immobilization stress + enriched environment group. ^a^indicates p < 0.05 vs. the control group, ^b^indicates p < 0.05 vs. the CEE group, and ^c^indicates p < 0.05 vs. the CIS group. One-way ANOVA test followed by Tukey post-hoc test was used for statistical analysis

The hippocampus was immunohistochemically stained with anti-LC3 antibody to clarify the localization of autophagosomes and assess the autophagy process in the neurons in CA1, CA3, and DG regions in all experimental groups. The strong immune positive cytoplasmic reaction was localized in numerous positively LC3 immuno-stained neuronal cells of CA1, CA3, and DG in the control group (Fig. 6A(a, e, i)) respectively. Additionally, in the CEE group, there were many positive LC3 immuno-stained neuronal cells with strong cytoplasmic reactions in all layers of CA1, CA3, and DG (Fig. 6A(b, f, j)) with statistically insignificant difference from rats of the control group (p value > 0.05).

While in the CIS group, few positive LC3 immuno-stained cells in all layers of CA1, CA3, and DG were seen (Fig. 6A(c, g, k)). Moreover, some positive LC3 immuno-stained cells in all layers of CA1, CA3, and DG with moderate cytoplasmic reactions were revealed in the (CIS + EE) group (Fig. 6A(d, h, l)). The results were validated by quantitative examination; the positive immunostained cells number in all layers of CA1, CA3, and DG showed a statistically significant difference between the CIS and CIS + EE groups (p value < 0.05) (Fig. 6B).

#### Electron Microscopic Results of Dentate Gyrus (DG) Region

##### Granular Neurons of the DG Region

**Fig. 7:**
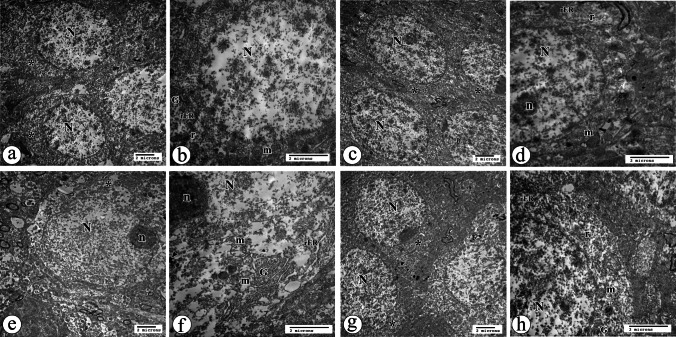
TEM of DG region of hippocampus tissue sections at level of granular neurons. **a** Control group showing large rounded euchromatic nucleus (N) and surrounded cytoplasm (*) (X 4800). **b** Control group showing the cytoplasm contains mitochondria (m), cisternae of Golgi (G), short strands of RER (rER), free ribosomes (r), and the euchromatic nucleus (N) (×10000). **c** CEE group showing adjacent granule neurons with a large rounded euchromatic nucleus (N) surrounded by its cytoplasm (*) (X 4800). **d** CEE group showing the cytoplasm contains mitochondria (m), short strands of RER (rER), free ribosomes (r), and the euchromatic nucleus (N) with prominent nucleoli (n) (×10000). **e** CIS group showing a large euchromatic nucleus (N) with a large prominent nucleolus (n) surrounded by a large electro-lucent cytoplasm (*) (X 4800). **f** CIS group showing part of a large euchromatic nucleus (N) with a prominent nucleolus (n) surrounded by rarified cytoplasm that contains wide cisternae of Golgi (G), elongated wide strands of RER (rER), and mitochondria with destructed cristae (m), in addition to lysosomal bodies (arrowhead) (×10000). **g** CIS + EE group showing large rounded euchromatic nucleus (N) surrounded by its cytoplasm (*) (X 4800). **h** CIS + EE group showing short strands of RER (rER), free ribosomes (r), cristae of Golgi (G), and some mitochondria (m) with destructed cristae. Notice part of its large euchromatic nucleus (N) (×10000)

The ultrastructure appearance of granule neurons in the control group (C) consisted of a large rounded euchromatic nucleus surrounded by a little amount of cytoplasm that contained mitochondria, cisternae of Golgi, short strands of RER, and free ribosomes (Fig. 7a, b). The similar findings were noticed in the granular neurons of the CEE group as the control (Fig. 7c, d).

While in the CIS group, the granular cells had damaging findings, such as large electro-lucent rarefied cytoplasm that contained wide cisternae of Golgi, elongated wide strands of RER, disorganized cristae structure, and lysosomal bodies (Fig. 7e, f). However, many granular neurons without any apparent pathological findings were observed in the CIS + EE group, except for a few mitochondria with destructed cristae (Fig. 7g, h).

##### Astrocytes of the DG Region

**Fig. 8:**
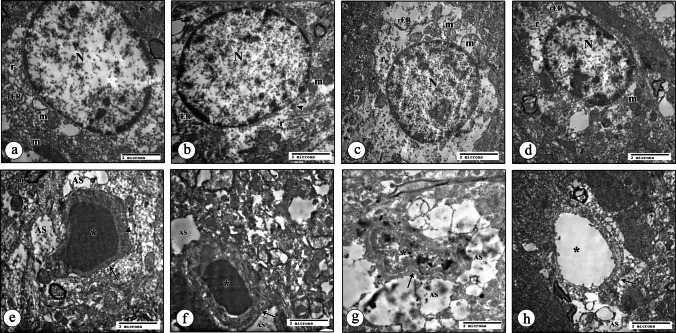
TEM of DG region of hippocampus tissue sections at level of astrocyte cells and blood capillaries (×10000). **a** Astrocyte cells of control group showing an oval nucleus (N) surrounded by electron-lucent cytoplasm containing mitochondria (m), short strands of RER (rER), free ribosomes (r), and lysosomes (arrowhead). **b** CEE group revealing an oval large nucleus (N), mitochondria (m), short strands of RER (rER), free ribosomes (r), and lysosome (arrowhead). **c** CIS group exhibiting an oval nucleus (N) contains few clumps of scattered and peripheral heterochromatin. The swollen electron lucent cytoplasm contains elongated strands of RER (rER), free ribosomes (r), and some mitochondria (m) with destructed cristae. **d** CIS + EE group presenting an oval large euchromatic nucleus (N) with clumps of heterochromatin, mitochondria (m), short strands of RER (rER), and free ribosomes (r) within the cytoplasm. **e** Blood capillaries of control group showing a wide lumen (asterisk). The endothelial cell contains electron-dense bodies (arrowhead) and is surrounded by regular basal lamina (arrow). Note the astrocytic process (As) and the myelinated nerve fibers (my) in the surrounding neuropil. **f** CEE group revealing an open lumen (asterisk) with a regular basal lamina (arrow) that is surrounded by the astrocytic processes (As). **g** CIS group exhibiting irregular narrow lumen (asterisk). The endothelial cell is surrounded by irregular basal lamina (arrow), and the astrocytic process (As) within the rarified wide surrounding neuropil. **h** CIS + EE group displaying wide patent lumen (asterisk). The endothelial cell is enclosed by irregular basal lamina (arrow) and surrounding astrocytic process (As)

The control and CEE groups showed more or less normal astrocyte cells (Fig. 8a, b). The astrocyte cells of the CIS showed some worsening findings, as swollen electron-lucent cytoplasm contained elongated strands of RER and some mitochondria with destructed cristae, as can be seen in (Fig. 8c). In contrast, CIS + EE group astrocytes showed no apparent pathological findings (Fig. 8d).

##### Blood Capillaries of the DG Region

Open wide lumens of blood capillaries, which were surrounded by regular basal lamina, were the common features in the control and CEE groups (Fig. 8e, f). While in the CIS group, blood capillaries revealed irregular narrow lumen and irregular basal lamina that were surrounded by rarified wide neuropil (Fig. 8g). The blood capillaries of the CIS + EE group were quite near to the control group (Fig. 8h).

### Correlation Analysis

There are significant positive correlations between BDNF, AQP4, GluA1, GluA2, LC3, and the number of crossed lines (r = 0.77, 0.45, 0.83, 0.58, 0.76; respectively); the number of central square entries (r = 0.48, 0.37, 0.70, 0.36, 0.55; respectively); time spent in the central square (r = 0.74, 0.42, 0.79, 0.54, 0.82; respectively) in the open field test; the discrimination index after 4 h (r = 0.79, 0.49, 0.86, 0.61, 0.84; respectively); and the discrimination index after 24 h (r = 0.75, 0.51, 0.87, 0.62, 0.82; respectively) in the novel object recognition test and significant negative correlations with the immobility time (r = − 0.68, − 0.49, − 0.87, − 0.54, − 0.82; respectively) in the tail suspension test.

There is a significant negative correlation between corticosterone level and BDNF, GluA1, GluA2, LC3 (r = − 0.78, − 0.90, − 0.59, − 0.83; respectively). In addition, BDNF has a significant positive correlation with GluA1, GluA2, and LC3 (r = 0.78, 0.61, 0.70; respectively), Figures can be seen in the supplement.

## Discussion

Chronic stress caused trouble in almost all body systems, including the central nervous system (Dolotov et al. 2022). It resulted in the accumulation of pathogenic proteins and free radicals, triggering cognitive impairment and neurodegeneration, especially in the hippocampus, the most vulnerable brain region to stress (Wei et al. 2019). The enriched environment (EE) is a promising non-invasive therapeutic method that improves neuronal structure (McCreary and Metz 2016), reduces anxiety, fear, and stress, and improves learning capacities compared to those maintained under standard conditions (Sakhaie et al. 2020).

Thus, the current research is designed to investigate the EE on CIS-induced cognitive, behavioral, molecular, and structural changes in adult male rats through the possible underlying mechanism. The CIS model was used in the current study, as it is the most widely used model, which is characterized by a combination of psychological and physical stressors by restricting the movement of animals and sorting them from their groups (Hafez et al. 2021).

Chronic immobilization stress (CIS) and its cognitive, behavioral, and structural changes were successfully induced in this study, as indicated by increased corticosterone level, decreased locomotor activity in OFT, reduced intermediate and long-term memory in the NOR test, increased immobility time in the tail suspension test, and structural changes in the hippocampus.

The present study offers a comprehensive evaluation of EE on hippocampal tissue in CIS-induced rats through physiological, biochemical, molecular, and histopathological assessments. At the physiological behavior evaluation level, the open field test is a behavioral test designed to assess rats' exploratory behavior and emotional responses in new environments (Liang et al. 2013). In the current work, the CIS group had a decreased number of crossed squares and central square entries and time spent in the central area in OFT compared to the control group, attributed to the fact that stress decreases locomotion and exploratory behavior (Liang et al. 2013; Shilpa et al. 2017; Thamizhoviya and Vanisree 2019) and induces depression-like behaviors (Hou et al. 2015; Wei et al. 2019).

EE exposure after CIS increased the locomotor activity as indicated by increased overall distance traveled in OFT compared to the CIS group, consistent with Thamizhoviya and Vanisree (2019). On the contrary, Shilpa et al. (2017) reported that EE exposure after CIS had no influence on CIS-induced decreased exploratory behavior in the OFT. This could be attributed to the difference in protocol used; Shilpa et al. (2017) applied chronic immobilization stress for 2 h/day for 10 days, followed by 2 weeks of exposure to EE 6 h/day.

The novel object recognition test is a widely used discrimination behavioral assay for studying several aspects of learning and memory (Lueptow 2017). In this study, intermediate and long-term memory were impaired in the CIS group. These findings are on the same track as the findings of Meng et al. (2013), who found that CIS leads to learning and memory deficits in the NOR test. Meanwhile, exposure to EE after CIS increased the discrimination index, indicating improvement of intermediate and long-term memory after EE exposure.

The tail suspension test is one of the most used models for evaluating depressive behaviors (Shinde et al. 2015). In the current work, CIS increased the immobility time in rats in TST, indicating depression. While EE exposure after CIS decreased immobility time in TST, demonstrating the favorable anti-depressive effect of the EE on animals exposed to prolonged stress (Xu et al. 2009; Wei et al. 2019; Vanisree and Thamizhoviya 2021).

The hypothalamo-hypophyseal system is a key hormonal response system to stress. Glucocorticoids are linked to a variety of mood and cognitive disorders (Stephens and Wand 2012). High cortisol levels can negatively impact brain areas involved in mood regulation, including the hippocampus, prefrontal cortex, and amygdala. Prolonged exposure to high glucocorticoid levels can cause structural changes in brain regions, including decreased volume and impaired function that are linked to the development of depressed symptoms (Correia et al. 2023).

The elevated level of corticosterone noted in the current study in the CIS group is in alignment with existing research (Thamizhoviya and Vanisree 2019; Ismail et al. 2024). Persistent stimulation of the HPA axis by chronic stress resulted in increased corticosterone levels, which leads to the depletion of glucocorticoid receptors and a progressive decline in the HPA axis' negative feedback mechanism (Xu et al. 2025).

In the current study, EE reduced the levels of corticosterone compared to the CIS group. This could be attributed to the ability of EE to normalize the HPA axis's reactivity and the ongoing impairment of the HPA axis' negative feedback mechanism (Morley‐Fletcher et al. 2003; Thamizhoviya and Vanisree 2019). EE increases both the number and activity of glucocorticoid receptors; this would increase the hippocampus's responsiveness to corticosterone activation, thereby enhancing the negative feedback mechanism (Smail et al. 2020). Glucocorticoid receptor upregulation is associated with enhanced cognitive functions and decreased anxiety-like behaviors, and this could be one of the mechanisms by which EE improved the behavioral changes induced by CIS in the current study (Shilpa et al. 2017).

Neuroprotective factor (BDNF) promotes survival, synaptic plasticity, and dendritic growth/branching in the nervous system and regulates the development of new neurons from neural stem cells (Bathina and Das 2015; Xu et al. 2021). Our work found that there was a decrease in BDNF levels in the hippocampus of CIS exposed rats. Previous research had shown that chronic and traumatic stress in animal models leads to increased DNA methylation of BDNF exon IV and decreased BDNF mRNA and protein expression (Roth et al. 2011; Niknazar et al. 2016, 2017; Zhao et al. 2020).

Taken together, stress-induced HPA axis hyperactivity leads to increased glucocorticoid levels, which decrease BDNF expression. BDNF can control the activity of this axis, potentially lowering glucocorticoid levels (Correia et al. 2023). This relationship between glucocorticoid and BDNF could clarify the decline of BDNF in the CIS group and restoration of its level after EE exposure.

The neurotrophic theory of depression suggests that depression is caused by low BDNF levels in the brain, which can be treated with antidepressants to alleviate symptoms and enhance BDNF levels (Rana et al. 2021). The reduction of BDNF observed in the current study could explain the reduction in the number of crossed lines, the number of central square entries, and the time spent in the central square observed in OFT. Moreover, it could explain the increase in immobility time observed in the current study in TST.

Moreover, exposure to EE after CIS led to increased BDNF levels in the hippocampus, which may explain the antidepressant effects of EE in the CIS model (Shilpa et al. 2017). Additionally, Xu et al. (2021) reported that EE enhanced learning and memory via enhancement of BDNF and its tyrosine kinase receptor type 2, which results in neurogenesis, proliferation, and differentiation of neurons and long-term potentiation. This could explain the improvement of memory and exploratory behavior seen in the CIS + EE group in the current study. Higher levels of BDNF in the hippocampus are directly associated with anxiolytic-like effects, according to the results of the Open Field Test (OFT). This indicates that rats with higher levels of BDNF showed less anxiety and better exploration. This improvement most likely occurs as a result of BDNF activating its receptor, TrkB (Stajic et al. 2019).

More strikingly, aquaporin-4 (AQP4) is the most plentiful aquaporin in neural precursor cells and perivascular endfeet of astrocytes, serving as a pseudo-lymphatic system in the brain, and is essential for the establishment of BBB stability (Iliff et al. 2012; Oklinski et al. 2016; Genel et al. 2021). Moreover, AQP4 plays an important role in neuronal multiplying, variation, and apoptosis (Kong et al. 2008; Kinoshita et al. 2009), cell migration, axonal growth, and synaptogenesis (Zheng et al. 2010).

Decreased the level of AQP4 in the rat hippocampus of CIS rats, indicating an impaired glymphatic system, which is essential for clearance of harmful substances from the brain. This is reliable with the conclusions of Xia et al. (2017) and Wei et al. (2019), who discovered that chronic unpredictable mild stress lowered AQP4 expression in the mice cortex. Yang et al. (2013a, 2013b) found that AQP4 depletion impairs hippocampal long-lasting increase in synaptic efficacy, and this could explain the impaired memory seen in the current study in the NOR test.

Preclinical AQP4 wild-type and knockout research revealed that exposure to stress or inflammation, as depression models, reduced AQP4 gene and protein expression in many brain areas, including the prefrontal cortex and the hippocampus, resulting in decreased neurogenesis and gliogenesis, as well as increased apoptosis and depressive-like behaviors (Genel et al. 2021).

Exposure to EE after CIS, in the current study, increased AQP4 level in the hippocampus. To our knowledge, no research investigates the effect of EE on stress-induced reduction in AQP4. Kong et al. (2014) showed that AQP4 knockout increases astrocyte vulnerability to corticosterone injury and aggravates corticosterone-induced diminishing of astrocytic functions, such as glutamate uptake and neurotrophin production, which inhibits neuroplasticity in the hippocampus. These findings give direct evidence that AQP4 has a great role in fundamental biological processes of mood change etiology, as evidenced in the current study by the decreased number of crossed lines and decreased quantity of central square entries and period consumed in the central square.

α-amino-3-hydroxy-5-methyl-4-isoxazolepropionic acid (AMPA) receptors are mostly located in the excitatory postsynaptic membrane (Yokoi et al. 2012). There are two types of AMPARs generated by combining their subunits: GluA1-containing AMPARs are Ca^2+^ permeable, while GluA2-containing AMPARs are Ca^2+^ impermeable (Diering and Huganir 2018). Changes in the number of subunits of AMPA receptors may have an effect on the activity and transmission of excitatory synapses. This dynamic mechanism is likely to play an important part in synaptic softness, which is thought to trigger components of knowledge and recall (Liang et al. 2013). Disruptions in AMPAR-mediated synaptic transmission in the hippocampus have been linked to stress reactions in both animal models and persons with depression (He et al. 2023).

Significant reductions in glutamate receptor expression in the hippocampus of CIS rats were observed; indicating disrupted glutamatergic transmission steady with previous outcomes of Hou et al. (2017), who reported that chronic unpredictable mild stress decreases GluA1 and GluA2 appearance in the hippocampus, which can reduce excitatory synaptic transmission in the hippocampus, leading to undesirable behaviors during chronic stress (Flowers et al. 2024).

Although preclinical and clinical research indicates that chronic stress reduces GluA1 levels in hippocampus synapses, there is conflicting evidence about changes in hippocampal GluA2 (He et al. 2023).

Elevated levels of both GluA1 and GluA2 expression in the hippocampus were observed in the CIS + EE group, which is consistent with the findings of Mlynarik et al. (2004) and Naka et al. (2005), who reported that the expression of GluA1 and GluA2, respectively, in the hippocampus was considerably greater in animals exposed to EE compared to control animals. Gao et al. (2023) found that EE exposure improved post-surgery sleep deprivation-induced cognitive impairments by upregulating BDNF and GluA1 expression. BDNF activation of TrkB rapidly up-regulates GluA1 and GluA2 protein levels in cultured hippocampus neurons by boosting transcription activity (Caldeira et al. 2007). Increased expression and activity of GluA1 and GluA2 subunits could be responsible for the anti-depressive and anti-stress effects of EE (Flowers et al. 2024). Thus, the improvement of stress-induced behavioral and cognitive changes by EE in the current study could be explained by the restoration of the level of BDNF and thereby increasing GluA1 and GluA2 levels.

At the histopathological level, we observed that the EE, when used alone, can preserve more or less the histoarchitectural and ultrastructural appearance of the hippocampus as the control group. Also, we noticed that the EE can protect the hippocampal structure from the damaging effects of CIS.

In the CIS group, the H&E-stained sections of the hippocampus revealed significant neuronal alterations in the CA1, CA3, and dentate gyrus regions. Huang et al. (2015) demonstrated that chronic restraint stress causes hippocampal CA1 and CA3 dendritic retraction and significantly reduced dendritic spine density in pyramidal neurons, which is possibly due to glucocorticoid elevation and hippocampal apoptosis, and correlates with spatial memory problems in male rats. Furthermore, Jiang et al. (2021) showed that in chronic restraint stress rats, the hippocampal CA1, CA2, and DG areas showed significant morphological alterations of neuronal cells with an increased Bax/Bcl-2 ratio and activated the pro-apoptotic proteins caspase-3 and 9 in the hippocampus, which resulted in neuronal cell loss. In addition, Thongrong et al. (2023) explained substantial neuronal injury in the CA1 and CA3 areas by increased reactive oxygen species (ROS) generation and lowered antioxidant levels.

Regarding the CIS + EE group, improvements in the histological and ultrastructural appearance of hippocampus CA1, CA3, and DG areas with many intact pyramidal neurons containing pale nuclei and many closely packed granule neurons with pale vesicular nuclei were observed in our study.

Pamidi and Nayak (2014) showed that EE exposure significantly increased the quantity of surviving neurons in CA1 and CA3 and reversed the reduction in hippocampal cell multiplying caused by chronic stress in anxious rats. Moreover, Kumar and his colleges (Kumar et al. 2018) found that the stressed rats exposed to EE had more surviving neurons in the CA3 hippocampal subfields than the stressed group, which was possibly due to alterations in the hippocampal BDNF expression.

Ultra-thin sections in the DG region in the CIS group showed that the DG granular cells and astrocytes had some deterioration findings, such as large electro-lucent rarified cytoplasm that contained damaged cisternae of Golgi, rough endoplasmic reticulum, some mitochondria with destroyed cristae, and irregular narrow blood capillaries that surrounded wide neuropil of the DG area, indicating severe tissue damage that enhances activation of the apoptotic pathway.

In line with prior electron microscopy studies, CIS rats were revealed ultrastructural damage in the hippocampus CA1, particularly mitochondrial damage or other dysfunction that severely impeded ATP synthesis, reduced primary neuronal capacity, and induced other alterations in cell structure and function disorder (Liang et al. 2013).

Astroglia are brain cells vital to the normal task and maintenance of neurons. Based on the provided search results, demonstrated astrocytic dysfunction in depression and chronic stress rodent models. These changes correlated with the behavioral deficits associated with long-lasting stress, which induces a progressive atrophy of cortical astroglial cells, potentially contributing to communication changes associated with stress-related illnesses (McEwen et al. 2016; Codeluppi et al. 2021).

Additionally, chronic stress can also impact hippocampal vasculature, leading to reduced cerebral blood flow, decreased angiogenesis, and diminishing of blood–brain barrier purpose. Chronic stress can lead to endothelial impairment and downregulation of pro-angiogenic growth factors and thickening of basal membranes, possibly affecting neuronal health and function (Albadawi 2025).

The ultrastructural appearance of the dentate gyrus (DG) in the CIS + EE group confirmed the light microscopic findings, which revealed many granular neurons and astrocytes without any apparent pathological findings, and the blood capillaries were quite nearby to the control group, which correlates to the improvement capacity of EE in neuronal survival.

Autophagy is a key mechanism that governs organismal homeostasis; when this balance is disrupted, pathogenic diseases can occur, such as major depressive disorder, dementia, cancer, liver disease, diabetes, cardiomyopathy, and autoimmune diseases (Gómez-Virgilio et al. 2022; Eladawy et al. 2025). Chronic stress can disrupt normal autophagy processes in the brain, and chronic disruption of autophagic flux could lead to a learning deficit (Chen et al. 2023).

LC3 is essential for the initiation of processing and extending the phagophore membrane and enhances the autophagosome-lysosome fusion, followed by lysosomal protease degradation of overwhelmed particles (Huang and Liu 2015).

The immunohistochemical analysis of the hippocampus in CIS rats revealed a significant decrease in the mean number of LC3 positive immune-stained cells. Quantitative data supported the qualitative observations, with a significant increase in the mean number of LC3 immuno-positive cells in the CIS + EE group compared to the CIS group was noted. Silva et al. (2019), who found that chronic stress reduced the levels of LC3 in the hippocampus, reported that activating the mammalian target of rapamycin (mTOR) pathway may contribute to inhibiting autophagic potential.

Research has shown that excess glucocorticoids result in dysregulation of autophagy (Wang et al. 2017; Ma et al. 2020). The machinery for stress-level glucocorticoids dysregulating autophagic processes is via mTOR, which is the key negative regulatory factor in autophagy induction and exerts an inhibitory effect on the autophagic activity (Li et al. 2020). Chen et al. (2013) and Puri and Subramanyam (2019) demonstrated that the neuroprotective influence of BDNF correlates with enhanced autophagy by upregulated LC3 and suppressed the Akt/mTOR/p70S6K pathway.

Takahashi et al. (2014) found that after 2 weeks of EE exposure, the expression of hippocampal LC3-II/β actin increased in the inescapable stress-treated group. Moreover, Xu et al. (2022) reported that EE attenuated the reduction of autophagy markers LC3 and beclin-1 induced by chronic unpredictable mild stress, as well as reduced inflammasomes and proinflammatory cytokines.

Despite the valuable findings obtained in the present study, some limitations should be acknowledged. Firstly; because sex hormones have been shown to have an impact on stress responsiveness, cognitive function, and hippocampus plasticity, the results cannot be applied to female rats because only adult male rats were used. To give a more thorough understanding of how each sex responds to environmental enrichment and chronic stress, future research involving both sexes and taking estrous cycle fluctuations into account is necessary. Secondly: The prefrontal cortex and amygdala, two other brain areas crucial for stress management and cognitive processing, were not assessed. Examining these fields could provide more information about the larger neurobiological processes that underlie both EE-mediated recovery and stress-induced dysfunction. Thirdly; other important signaling pathways related to neuroinflammation, oxidative stress, apoptosis, and synaptic remodeling were not explored. Comprehensive molecular profiling could further clarify the mechanistic basis of EE-mediated neuroprotection and finally the study assessed outcomes at a single time point following chronic exposure. Longitudinal studies evaluating both short- and long-term effects of EE are needed to determine the persistence and durability of its protective effects.

## Conclusions

Collectively, based on correlation results, these data could suggest that chronic stress and high corticosterone levels reduce BDNF, which decreases levels of GluA1, GluA2, AQP4, and LC3 expression, resulting in histopathological hippocampal tissue deterioration and thereby the behavioral and cognitive changes observed in the existing study. Applying EE in CIS rats greatly alleviates these damaging alterations and could be explained by the normalization of corticosterone level and the restoration of the level of BDNF, thereby increasing GluA1, GluA2, AQP4, and LC3 levels, resulting in improvement in the histological and ultrastructural appearance of the hippocampus, which is reflected on the function of the hippocampus.

## Data Availability

The datasets generated during and/or analyzed during the current study are available from the corresponding author on reasonable request.

## Abbreviations

EE: Enriched environment
CIS: Chronic immobilization stress
BDNF: Hippocampal brain-derived neurotrophic factor
AQP4: Aquaporin 4
GluA1, GluA2: Glutamate receptors
LC3: Microtubule-associated protein -light chain 3
